# Anterior cervical discectomy and fusion with a zero-profile VA spacer device: a clinical and radiological study with two-year follow-up

**DOI:** 10.1186/s13018-024-04539-9

**Published:** 2024-01-11

**Authors:** Hai-Hong Zhao, Hao-Wei Xu, Shan-Jin Wang, Tao Hu, De-Sheng Wu

**Affiliations:** Department of Spine Surgery, Shanghai East Hospital, School of Medicine, Tongji University, Shanghai, 200092 People’s Republic of China

**Keywords:** Zero-P VA, Dysphagia, Cervical spine, Cervical alignment, Spinal fusion

## Abstract

**Study design:**

A retrospective study.

**Objective:**

The aim of this study was to compare clinical and radiological outcomes of the anterior cervical discectomy and fusion (ACDF) with a novel zero-profile variable-angle (Zero-P VA) spacer and a traditional poly-ether-ether-ketone (PEEK) cage and plate system in cases pertaining to cervical radiculopathy/myelopathy.

**Summary of background data:**

There are two conventional types of ACDF procedures aimed at treating symptomatic cervical spondylosis. The first one involves an uninstrumented “stand-alone” approach utilizing bone graft/cage, while the second incorporates bone graft/cage in conjunction with a front plate positioned before the vertebral bodies. Both procedures have their own inherent advantages and disadvantages. The Zero-P VA spacer, however, represents a unique synthesis by amalgamating the advantages of both traditionally typical procedures. Notably, this spacer can potentially circumvent the issue related to prevertebral soft-tissue disturbance and reduce the occurrence of dysphagia.

**Methods:**

Using our surgical database, the authors systematically conducted a retrospective analysis encompassing all patients who underwent single-level ACDF between January 2018 and January 2019, with a minimum two-year follow-up. Patients either received a Zero-P VA implant or PEEK cage coupled with plating. The Japanese Orthopedic Association (JOA) score and Visual Analogue Scale (VAS) for arm and neck pain were documented. Dysphagia was evaluated using the Eating Assessment Tool-10 (ETA-10). Additional parameters such as cervical alignment, fusion rate and the incidence of postoperative complications were assessed.

**Results:**

According to the outcomes of the statistical analysis, there was no substantial disparity that emerged in the advancements observed in the JOA and VAS metrics between the two study cohorts. Noteworthy, however, the ETA-10 scores were statistically significantly reduced in the Zero-P VA group compared to the cage and plating group (*p* < 0.05). At the final follow-up, there were no statistically significant differences in the height of the operated segment, Cobb angle of the fused segment, C2–C7 Cobb angle and fusion rate between the two groups (*p* > 0.05). However, postoperative complications were slightly lower in patients with the Zero-P VA group (7.69%) as compared to the cage and plating group (16.67%).

**Conclusion:**

The clinical outcomes observed with the Zero-P VA spacer used for single-level ACDF were found to be satisfactory. The performance of this device is comparable or even superior to the traditional cage and plating method in preventing postoperative dysphagia and mitigating potential complications associated with the use of a plate.

## Introduction

Anterior cervical discectomy and fusion (ACDF) is a standard surgical procedure for cervical radiculopathy/myelopathy cases [1]. While new dynamic reconstruction technologies have made significant progress, it is important to note that spinal fusion is still the primary treatment option for the majority of degenerative disk cases [2]. The anterior approach has indeed been widely used in the treatment of cervical degenerative disk diseases for several decades [3]. However, there are no generally accepted techniques to achieve it to date. Currently, there were two typical ACDF procedures: (1) stand-alone autologous bone graft or cage and (2) bone graft or a cage anchored with a plate in front of the vertebral bodies [4, 5]. Both techniques have their own advantages and potential disadvantages. The commonly mentioned drawbacks of these techniques include postoperative dysphagia associated with plating and reduced stability with cage subsidence when using a stand-alone implant, which are often discussed in relation to these techniques [2]. Low-profile angle-stable spacer is a novel concept implant that promises the potential to avoid the drawbacks of both traditional techniques [6]. In particular, it can provide immediate stability of an index level as compared to the stand-alone cage; meanwhile, it may avoid the problem with dysphagia associated with plating concept [6].

The objective of this study was to compare clinical and radiological outcomes of the zero-profile variable-angle (Zero-P VA) spacer and traditional polyetheretherketone (PEEK) cage and plating system after ACDF in the cervical radiculopathy/myelopathy patients.

## Materials and methods

**Fig. 1:**
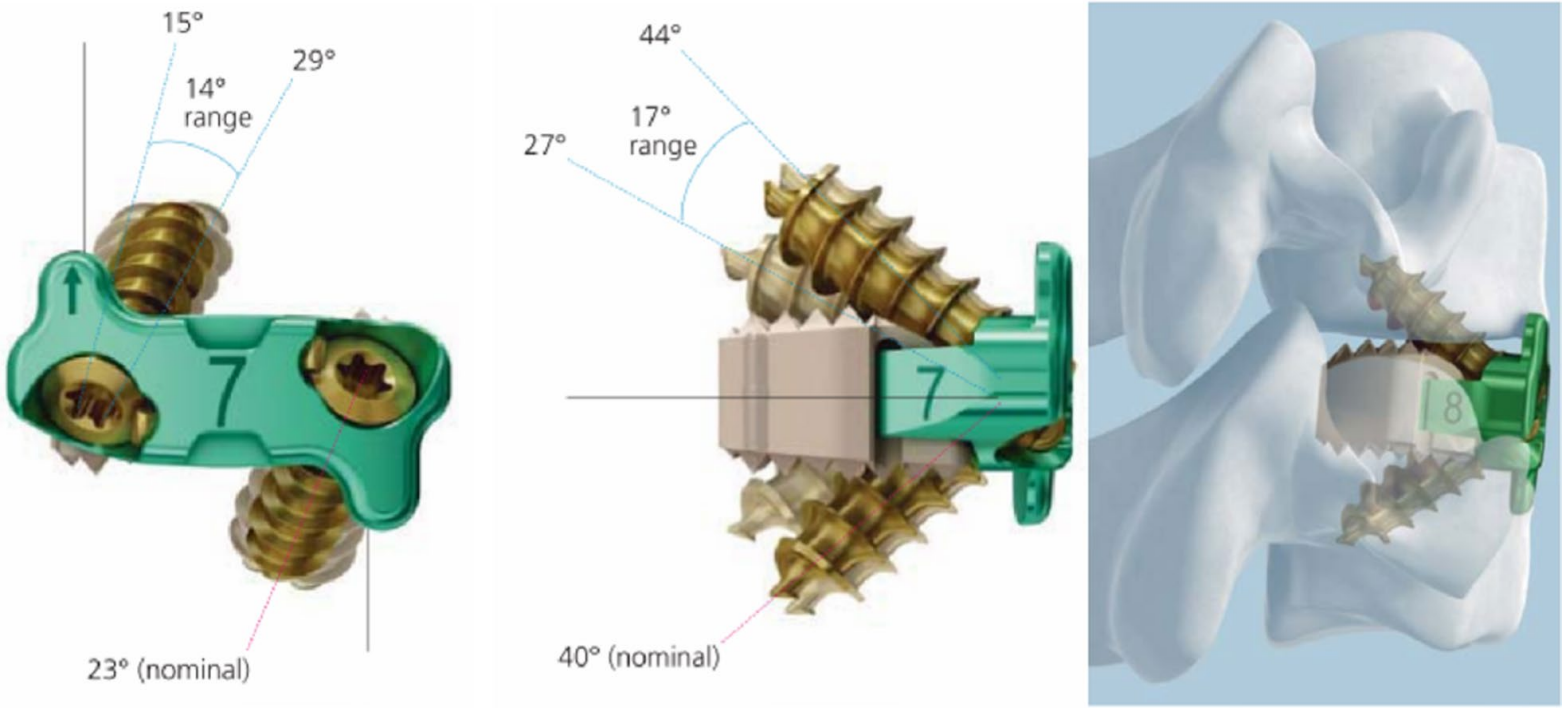
Images of ZERO-P VA instruments and implants

This retrospective study received approval from our hospital ethics committee and involved 43 patients (17 were females, 26 were males) presenting with cervical myelopathy and/or radiculopathy failing to respond to conservative treatment. The study groups have been differentiated based on the internal fixation materials that have been employed: the Zero-P VA (Fig. 1) was implanted in 13 patients (Group A), while 30 patients were treated with traditional plate and cages (Group B). Inclusion and exclusion surgery criteria adhered to widely accepted indications and contraindications for cervical spondylosis listed in Table 1. All surgical procedures were conducted under general anesthesia and were performed by the same senior surgeon utilizing the standard ACDF technique. In brief, after thorough decompression and removal of the cartilaginous endplate, the appropriate Zero-Profile Variable-Angle (DePuy Synthes Spine, Raynham, MA, US) device was selected after trialing and then implanted into target intervertebral disk level in Group A. In Group B, suitable intervertebral cage (Cervios, DePuy Synthes Spine, Raynham, MA, US) was implanted and pre-bent locking plate (Slim Loc Anterior Cervical Plate System; DePuy Synthes Spine, Raynham, MA, US) of suitable curvature and length was placed. Locking screws were placed for fixation, and correct positionings of the implants were confirmed on lateral fluoroscopy intraoperatively. After surgery, all patients received consistent medications, including analgesic (Parecoxib, Pharmacia and Upjohn Company, 40 mg/qd), methylprednisolone (Pfizer Manufacturing Belgium NV, 40 mg/qd), and gastric mucosal protectant (Pantoprazole, Takeda GmbH, Germany, 40 mg/qd) for two days. After discharge, all patients used NSAIDs (Celecoxib Capsules, Pfizer Pharmaceuticals LLC, 200 mg/bid) and Mecobalamin tablets (Eisai China pharmaceutical co. LTD, 0.5 g/tid) for one month. Standard demographic data, surgical data, and peri-operative complications were recorded. Patients typically receive regular follow-up appointments in the out-patient clinic at 1, 3, 12, 24 months after surgery. During therapeutic process and follow-up, patients were asked to complete the Japanese Orthopedic Association (JOA) and Visual Analogue Score (VAS) questionnaire to assess the overall amount of disability and degree of pain in neck and upper limbs caused by their cervical spine pathology, and the incidence of dysphagia was assessed using the Eating Assessment Tool (EAT-10) [7]. The functional and radiological assessments were independently conducted by two surgeons at different time points: preoperatively, 2 days postoperatively, 1 month postoperatively, 3 months postoperatively, 12 months postoperatively, and 24 months postoperatively. We compared preoperative and final follow-up plain radiographs, computed tomography and magnetic resonance images for all patients.

**Table 1:** Patient selection criteria

Criteria
Inclusion
Age 30–70 years
Failure of nonsurgical treatments for 6 weeks
Symptomatic cervical disk disease between C3 and C7 with neck or arm pain and/or
Functional/neurologic deficit confirmed by imaging (X-ray, CT and MRI)
Exclusion
Previous surgery at the index level
Patients having contraindication for anesthesia
Traumatic cervical subluxation
Systemic or local infection
Active rheumatoid arthritis or any other medical condition(s) that would increase surgical risk or interfere with normal healing
Known history of osteoporosis
Previous known allergy to the materials contained in the device
History of any invasive malignancy

Radiologic evidences of complications were determined using the following pre-established criteria: new anterior osteophyte formation or enlargement of existing osteophytes, cage subsidence and screw loosening. Clinical complications, such as wound infection, hematoma, severe dysphagia and prolonged drainage, were recorded. Postoperative X-rays and CT scan were obtained during follow-up to assess the radiographic parameters and fusion rates. Radiographic fusion was confirmed using the interspinous process method [8]. Before operation and during postoperative follow-up, the sagittal profile of the cervical spine was assessed using the Cobb angle, and C2–C7 Cobb angle was measured between the lower endplate of the second cervical vertebra and the seventh cervical vertebra. The Cobb angle of the fused segments was measured by drawing two lines connecting the upper endplate of the cranial vertebra and the lower endplate of the caudal vertebra [9].

Statistical analysis was performed using SPSS Version 19.0 (SPSS Inc., Chicago, IL, US). The Student’s t test and the Chi-square test were used to analyze differences between two groups. A *p* value < 0.05 was considered statistically significant.

## Results

The characteristics of the study are detailed in Table 2. In group A, the drainage tube removal time was 1.00 ± 0.00 days, and in group B for 1.2 ± 0.41 days, with a statistically significant difference (*p* = 0.012). However, the average operative time, intraoperative blood loss, length of stay and volume of drainage were not statistically significant between two groups (*p* > 0.05).

**Table 2:** Demographics of the subjects

Variables	Group A (Zero-P VA)	Group B (Plate and Cage)	*p*
Patient no.	13	30	
Sex (male/female)	5/8	12/18	0.925
Age (year)	59.15 ± 13.44	54.33 ± 12.61	0.265
Operation time (min)	83.92 ± 12.89	91.03 ± 16.18	0.169
Intraoperative blood loss (mL)	65.00 ± 30.14	57.33 ± 29.47	0.441
Follow-up time (month)	12.31 ± 1.18	12.13 ± 2.47	0.811
Length of stay (day)	9.31 ± 6.26	8.50 ± 4.19	0.808
Volume of drainage (ml)	52.77 ± 36.33	40.17 ± 24.44	0.269
Drainage tube removal time (day)	1.00 ± 0.00	1.2 ± 0.41	0.012
*Level of treated segments*			
C3–C4	5	2	
C4–C5	1	3	
C5–C6	4	18	
C6–C7	3	7	

All patients experienced alleviation of symptoms after the surgery. The mean JOA score increased significantly from 9.23 ± 1.36 points before surgery to 13.31 ± 1.03 points at the two-year follow-up in group A and from 8.87 ± 1.85 points to 12.93 ± 2.02 points in group B (*p* < 0.001). The mean neck VAS (3.85 ± 1.14 VS 1.77 ± 1.17) and arm VAS score (2.31 ± 0.95 VS 0.54 ± 0.66) decreased after operation in groups A (*p* < 0.001). The mean neck VAS and arm VAS of group B (3.53 ± 1.46 VS 1.70 ± 1.21, 2.00 ± 1.64 VS 0.77 ± 0.86) also significantly decreased after surgery (*p* < 0.001). However, there were no significant differences between two groups before and after the operation (Table 3). There were no dysphagia-related complaints preoperatively amongst patients. At 48 h postoperatively, a total of 21 patients complained of dysphagia and the ETA scores were higher in group B as compared to group A (12.27 ± 4.025 VS 9.23 ± 2.89, *p* = 0.019). At the 1-year follow-up, the symptoms of dysphagia had been resolved completely, whereas Group A was slightly better as compared to Group B until 1-year follow-up (Table 3).

**Table 3:** Clinical outcomes

	Group A (Zero-P VA)	Group B (Plate and Cage)	*p*
*JOA score*			
Preoperative	9.23 ± 1.36	8.87 ± 1.85	0.528
2-year follow-up	13.31 ± 1.03*	12.93 ± 2.02*	0.531
*VAS neck score*			
Preoperative	3.85 ± 1.14	3.53 ± 1.46	0.496
Postoperative	1.77 ± 1.17*	1.70 ± 1.21*	0.862
2-year follow-up	0.85 ± 1.07*	0.9 ± 0.96*	0.871
*VAS arm score*			
Preoperative	2.31 ± 0.95	2.00 ± 1.64	0.445
Postoperative	0.54 ± 0.66*	0.77 ± 0.86*	0.398
2-year follow-up	0.08 ± 0.28*	0.23 ± 0.50*	0.200
*EAT-10*			
Preoperative	0.31 ± 0.48	0.2 ± 0.41	0.455
Postoperative	9.23 ± 2.89*	12.27 ± 4.03*	0.019
1-month follow-up	5.00 ± 2.45*	7.8 ± 3.33*	0.009
3-month follow-up	3.38 ± 1.61*	5.53 ± 3.10*	0.023
1-year follow-up	1.77 ± 1.59*	2.87 ± 1.78*	0.062
2-year follow-up	1.38 ± 1.04	2.53 ± 2.19	0.080
Postoperative complications	7.69% (1/13)	16.67% (5/30)	0.435
Hoarseness	1	2	
C5 palsy	0	1	
Screw loosening	0	2	

^*^p < 0.05, compared with the previous group

*JOA* Japanese Orthopaedic Association; *EAT* Eating Assessment Tool

**Fig. 2:**
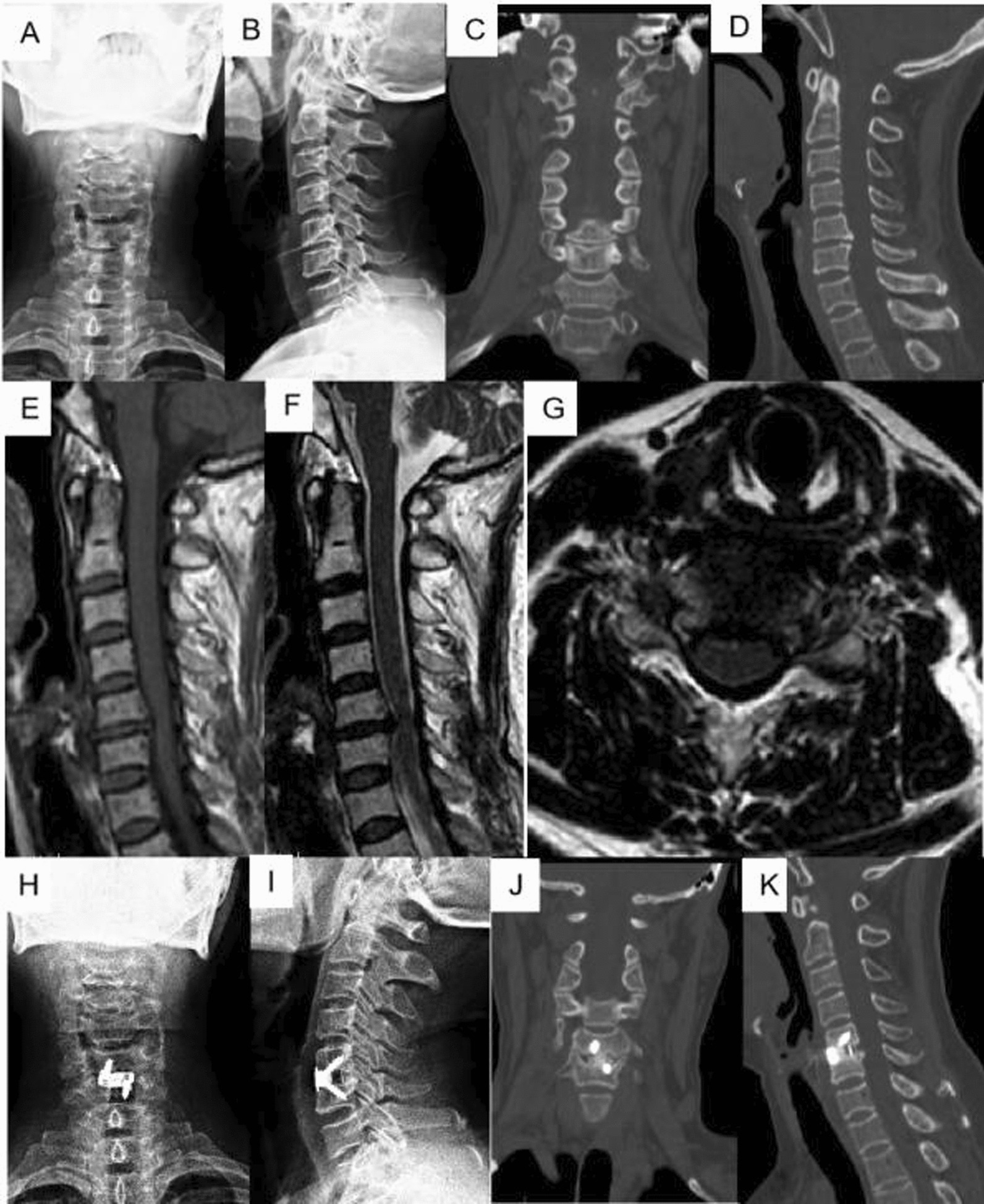
The patients underwent ACDF with ZERO-P VA internal fixation system. **A** Anteroposterior and **B** lateral of standing cervical X radiographs. **C**, **D** CT in 3D-sagittal reconstruction; **E**, **F** T1- weighted and T2-weighted MRI in sagittal plain, **G** MRI in axial plain of C5/6. Postoperative 6 months, **H** anteroposterior and **I** lateral of standing cervical X radiographs; **J**, **K** CT in 3D-sagittal reconstruction

**Fig. 3:**
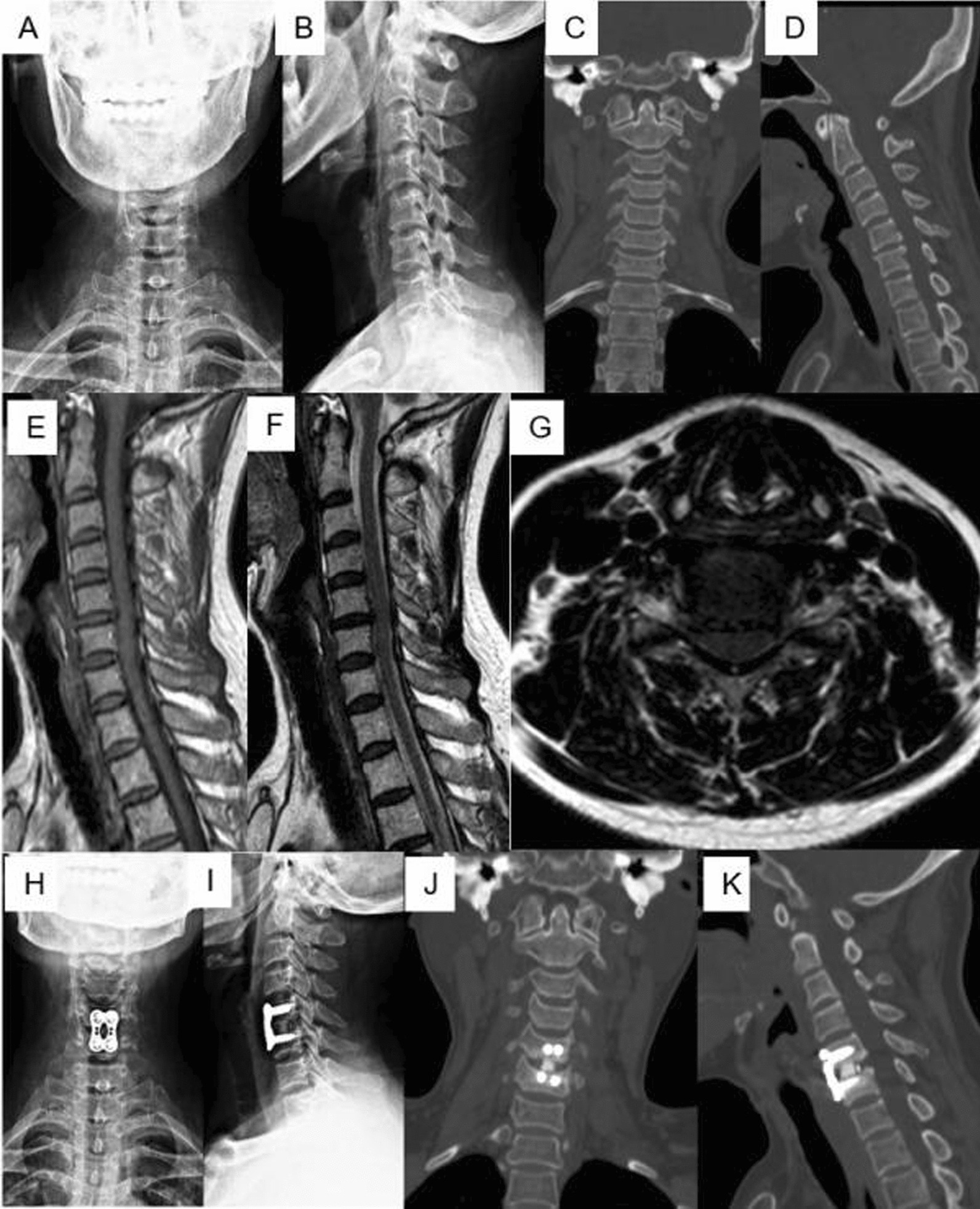
The patients underwent ACDF with plate and cage internal fixation system. **A** anteroposterior and **B** lateral of standing cervical X radiographs. **C**, **D** CT in 3D-sagittal reconstruction; **E**, **F** T1- weighted and T2-weighted MRI in sagittal plain, **G** MRI in axial plain of C5/6. Postoperative 6 months, **H** anteroposterior and **I** lateral of standing cervical X radiographs; **J**, **K** CT in 3D-sagittal reconstruction

**Fig. 4:**
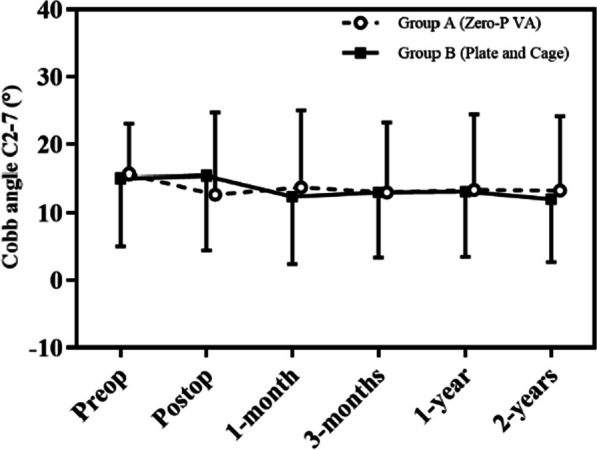
Development of C2–C7 Cobb angle during follow-up. Preop indicates preoperative; Postop, postoperative, 1 month, 3 months,1 year and 2 years

**Fig. 5:**
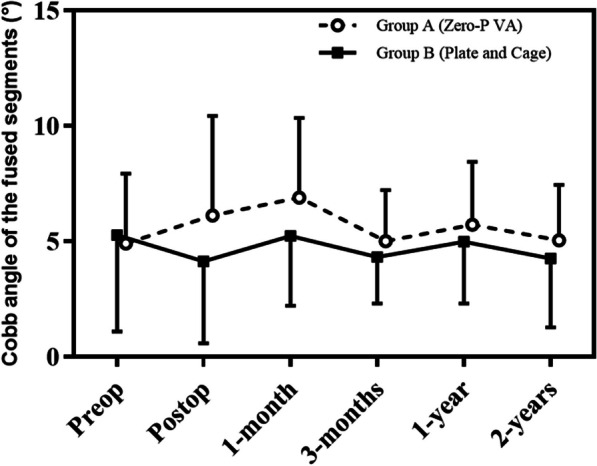
Cobb angle of the fused segments during follow-up. Preop indicates preoperative; Postop, postoperative, 1 month, 3 months,1 year and 2 years

Preoperative radiographs were evaluated by two independent, experienced spine surgeons, and postoperative imaging showed thorough decompression and satisfying internal fixation for both patient groups (Figs. 2, 3). The mean Cobb angle between C2 and C7 was comparable between two groups, and no significant difference was found preoperatively (15.71 ± 7.37 VS 15.41 ± 10.49, *p* = 0.927). There was no statistical difference on preoperative mean Cobb angle of the fused segments between two groups (4.90 ± 3.03 VS 5.27 ± 4.17, *p* = 0.773) (Table 4). At the two-year follow-up, the mean C2–C7 Cobb angle was 13.30 ± 10.83 degrees in group A and 12.00 ± 9.32 degrees in Group B (*p* = 0.691), and the mean Cobb angle of the fused segments was 4.98 ± 2.65 degrees in group A and 4.10 ± 2.83 degrees in Group B (*p* = 0.343). There were no obvious increases on the mean C2–C7 and fused segment Cobb angles for the two groups after the surgery and during the follow-up period (Figs. 4, 5). There were no statistical differences on preoperative and postoperative mean height of the fused segments between two groups (*p* > 0.05). Three patients experienced hoarseness occurred after surgery and completely resolved after one year. There was one patient who experienced left deltoid muscle weakening which might be due to C5 palsy, and recovered 3 months after the surgery. In addition, screw loosening occurred in one patient, but he did not have any clinical symptoms. There were no occurrences of postoperative wound infection, cerebrospinal fluid leakage, or hematoma. The fusion rate at the 2 years was 92.31% in group A and 90.00% in the group B, and there was no statistically difference between two groups. No patient received any revision surgery (Table 3).

**Table 4:** Radiologic outcomes

	Group A (Zero-P VA)	Group B (Cage and Plate)	*p*
*Height of operated segment*			
Preoperative	34.42 ± 4.06	34.32 ± 4.55	0.942
Postoperative	35.43 ± 3.19	36.33 ± 4.36	0.506
2-year follow-up	35.31 ± 4.78	36.35 ± 4.16	0.474
*Cobb angle C2–7 (°)*			
Preoperative	15.71 ± 7.37	15.41 ± 10.49	0.927
Postoperative	12.68 ± 12.07	15.44 ± 11.32	0.475
2-year follow-up	13.30 ± 10.83	12.00 ± 9.32	0.691
*Cobb angle of fused segment (°)*			
Preoperative	4.90 ± 3.03	5.27 ± 4.17	0.773
Postoperative	6.12 ± 4.31	4.13 ± 3.55	0.121
2-year follow-up	4.98 ± 2.65	4.10 ± 2.83	0.343
*Fusion rate (%)*			
6-month follow-up	84.61% (11/13)	83.33% (25/30)	0.917
1-year follow-up	92.31% (12/13)	86.67% (26/30)	0.858
2-year follow-up	92.31% (12/13)	90.00% (27/30)	0.811

## Discussion

The initial imperative for immediate postoperative stability prompted the development of an anterior plating system for ACDF [5]. Subsequently, advancements in the plating system have improved from static plates to the dynamic plates, limiting stress-shielding and thereby enhancing conditions for interbody spinal fusion [10]. Despite the reduction in the profile of the current plating system compared to that of first generation, they still interfered with anterior vital structures in front of the anterior cervical spine and potentially resulted in intraoperative and postoperative complications, such as esophageal perforation, tracheal and carotid arteries injury [11]. Furthermore, the prevalence rates of screw and plate fracture, of screw loosening, and of implant displacement are apparently high and have been posing challenges for both patients and surgeons [12]. Moreover, dysphagia is a consistent postoperative symptom that has been a frequent complain among patients, particularly in the early follow-up period [13, 14]. From our collected data, most of patients complaining about dysphagia had fully recovered within three months, and there has been no lingering complaints regarding swallowing after 6 months. These results are consistent with previous studies [15, 16], whereas the exact pathophysiologic cause for this symptom still remains unknown. In addition, a systematic review of anterior cervical spine surgery had reported dysphagia-related occurrences of up to 5.3% [17]. There was a systematic review, and meta-analysis illustrated that ACDF with zero-profile fixation was better than anterior cervical plate fixation regarding the incidence of postoperative dysphagia (P < 0.05), which further demonstrates the robustness of our study and substantiates the distinctive benefits of Zero-P fixation in mitigating postoperative dysphagia [18]. However, an alternate review reported no significant difference in dysphagia incidence between ACDF with and without anterior plate fixation, suggesting the need for more studies to elucidate the factors influencing dysphagia [19].

Nevertheless, it is important to acknowledge that no plating system is devoid of complications. An analysis of angle-stable systems has indicated failure rates of up to 22.8%, although most studies report a lower incidence of such complications [17]. The presence of a plate in the anterior cervical spine and the contact between the plate and the esophagus were recognized to be plausible inducements, which led to dysphagia post-operation, and the reported incidence of this complication ranges from 30% in the initial 3 months post-operation to 13–21% at 1 year [13, 14, 20]. This perspective could be supported by the significantly lower occurrence of postoperative dysphagia in patients undergoing stand-alone cervical arthroplasty compared to those treated with adjunctive cervical plating [21]. Additionally, the presence of a plate may potentially expedite degenerative alterations in adjacent segments [22].

Interbody cages are strategically designed for stand-alone implantation without additional anchoring or fixation. While this treatment method has gained widespread acceptance, it is crucial to note that there are potential drawbacks associated with this approach. A primary disadvantage of using an unanchored cage for anterior cervical fusion surgery is the diminished extension stability, which can lead to issues such as the cage sinking or the development of segmental kyphosis in the operative level [23]. Scholz et al. conducted an in vitro study to evaluate the instant biomechanical stability obtained in single level treated with the zero-profile (Zero-P) spacer [24]. There was a lower stability of the Zero-P device in flexion and extension compared with cages with a locking plate, but the differences were not deemed statistically significant. In addition to this, there were no significant differences between the groups in lateral flexion and rotation. The Zero-P spacer incorporates a plate and screw system, eliminating the inherent disadvantage of stand-alone cages, which is extension instability. A previous study has reported the use of Zero-P as internal fixation after a traumatic subaxial cervical disk injury, which might prove the biomechanical stability of this new device in vivo [25].

In this study, at the 6-week follow-up visit, the physiological load displayed a significant decline in the Cobb angle values of the fused segment in the group with the Zero-P spacer. Apart from the axial load, it was likely attributed to the aforementioned lower biomechanical stability of the implant in flexion and extension. In contrast, the same mechanism resulted in an increase in the Cobb angle values of the fused segment in patients with plate stabilization, where the physiological load of the segment opposed tension on the anterior spine caused by the plate, which corresponded to the maximum Cobb angle values of the fused segment measured at the 6-week postoperative mark. During the six-week postoperative follow-up period, both groups exhibited a decrease in the Cobb angle of the fused segment. However, no statistically significant differences were noted between the two monitored groups for the duration of the remaining follow-up period. It was published that mono- or bi-segmental cervical surgery did not have a significant impact on the complex sagittal profile of the cervical spine, even in instances where cage subsidence occurred, leading to progressive segmental kyphosis. This observation was consistent with the findings of our patient group, which also demonstrated that the complex sagittal profile of the cervical spine, as indicated by the Cobb angle measurement of C2–C7, did not represent any significant changes in either group during the postoperative progress.

The subsidence of stand-alone cages, whether accompanied by subsequent segmental kyphosis or not, was widely recognized as one of the major disadvantages of this type of cervical stabilization. Song et al. [4] compared the stand-alone cage technique and the technique of the cage with a locking plate and reported 32.3% of subsidence with unanchored cages against 9.7% in the group with the locking plate. We evaluated the degree of height reduction in the treated segments and observed no statistically significant difference in the degree of height reduction between the two groups under investigations.

Song et al. proposed that radiographs magnified 150% could be used to measure the interspinous motion to evaluate the state of anterior cervical fusion [26]. In this study, the statistically significant differences between the two groups were not found in the mean Cobb angle of C2-C7 and the fused segments preoperatively. This indicated the fusion level was stable, which might be the reason why there was no significant difference in fusion rate. In addition, we did not find a significant difference in the incidence of dysphagia during the follow-up. Although a lower incidence of dysphagia was found in those patients treated with Zero-P implant, this study lacked sufficient power to statistically demonstrate the superiority of the Zero-P group over the cage and plate group for dysphagia. Both approaches led to a significant reduction in pain in our patients, as assessed by the neck disability index. The same results were found in the patients’ overall satisfaction with follow-up at 2 years according to the criteria proposed by Odom [27]. At the same time, prospective randomized trials with more patients and longer follow-ups were necessary to confirm these observations.

### Study limitations

To validate these observations, multi-center prospective randomized trials involving larger cohorts and extended follow-up periods are imperative.

## Conclusions

The clinical outcomes observed with the Zero-P VA spacer used for single-level ACDF were found to be satisfactory. The performance of this device is comparable or even superior to the traditional cage and plating methods in preventing postoperative dysphagia and mitigating potential complications associated with the use of a plate.

## Data Availability

The datasets generated and/or analyzed during the present study were available from the corresponding author upon reasonable request.

## Abbreviations

ACDF: Anterior cervical discectomy and fusion
Zero-P VA: Zero-profile variable-angle
PEEK: Poly-ether-ether-ketone
JOA: Japanese Orthopedic Association
VAS: Visual Analogue Scale
ETA-10: Eating Assessment Tool-10
Zero-P: Zero-profile
CT: Computed tomography
